# Supplementary material for: Factors influencing estimates of HIV-1 infection timing using BEAST
Source: PLoS Comput Biol. 2021 Feb 1;17(2):e1008537. doi: 10.1371/journal.pcbi.1008537 (PMC7877758; doi:10.1371/journal.pcbi.1008537)
Supplement: S1 Table — n denotes the number of participant datasets. Negative estimates denote days prior to diagnosis. (DOCX) [file pcbi.1008537.s001.docx]

|  |  |  |  |  | Proportion of participants with an estimate: | | | |
| --- | --- | --- | --- | --- | --- | --- | --- | --- |
| founder | gene | n | median | IQR | after diagnosis | within 1 week | within 2 weeks | within 4 weeks |
| single | vpu | 9 | -5 | (-8,0) | 0.33 | 0.33 | 0.67 | 0.67 |
| single | vpr | 10 | -2 | (-9,3) | 0.40 | 0.30 | 0.50 | 0.50 |
| single | tat | 20 | 1 | (-6,3) | 0.55 | 0.25 | 0.40 | 0.40 |
| single | rev | 18 | -6 | (-7,2) | 0.39 | 0.33 | 0.50 | 0.56 |
| single | vif | 15 | -5 | (-8,-2) | 0.13 | 0.60 | 0.67 | 0.73 |
| single | nef | 22 | -4 | (-6,2) | 0.36 | 0.41 | 0.64 | 0.64 |
| single | gag | 21 | -6 | (-9,-4) | 0.10 | 0.48 | 0.71 | 0.86 |
| single | env | 24 | -6 | (-9,-2) | 0.12 | 0.54 | 0.79 | 0.88 |
| single | pol | 23 | -17 | (-40,-5) | 0.04 | 0.30 | 0.43 | 0.65 |
| single | genome | 25 | -10 | (-20,-5) | 0.08 | 0.36 | 0.52 | 0.80 |
| multiple | vpu | 11 | -89 | (-295,-20) | 0.09 | 0.09 | 0.09 | 0.27 |
| multiple | vpr | 9 | -292 | (-321,-9) | 0.11 | 0.11 | 0.33 | 0.33 |
| multiple | tat | 11 | -5 | (-47,0) | 0.27 | 0.36 | 0.45 | 0.45 |
| multiple | rev | 10 | -9 | (-41,-1) | 0.30 | 0.20 | 0.30 | 0.40 |
| multiple | vif | 9 | -66 | (-932,-8) | 0.00 | 0.22 | 0.44 | 0.44 |
| multiple | nef | 11 | -16 | (-60,-4) | 0.09 | 0.36 | 0.36 | 0.55 |
| multiple | gag | 10 | -141 | (-266,-17) | 0.00 | 0.20 | 0.30 | 0.30 |
| multiple | env | 9 | -200 | (-689,-25) | 0.22 | 0.00 | 0.00 | 0.11 |
| multiple | pol | 8 | -184 | (-371,-105) | 0.00 | 0.12 | 0.25 | 0.25 |
| multiple | genome | 9 | -225 | (-356,-3) | 0.22 | 0.11 | 0.11 | 0.11 |
