# Supplementary figures and images for: Factors influencing estimates of HIV-1 infection timing using BEAST
Source: PLoS Comput Biol. 2021 Feb 1;17(2):e1008537. doi: 10.1371/journal.pcbi.1008537 (PMC7877758; doi:10.1371/journal.pcbi.1008537)

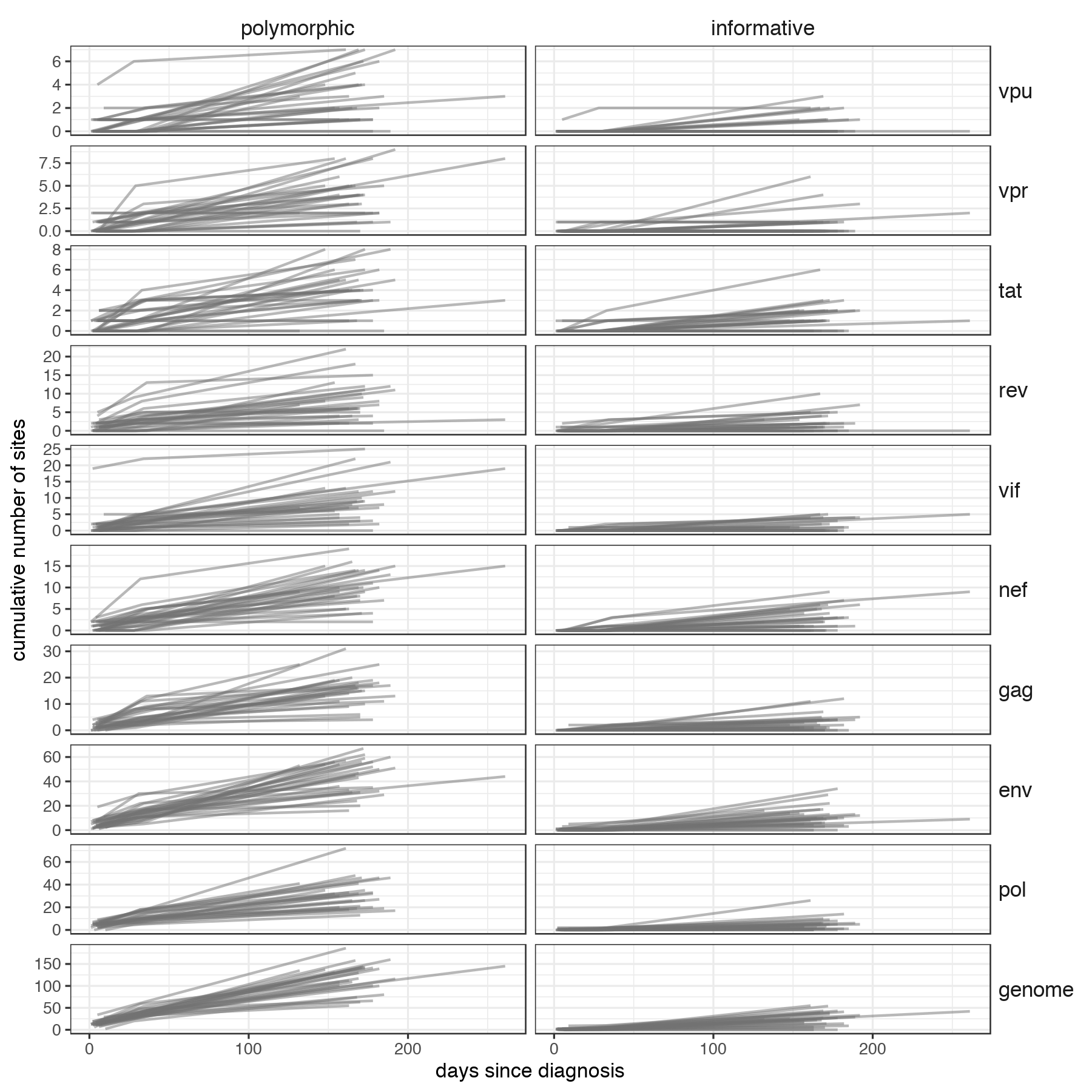

Supplement: S1 Fig — At each sampling time point, we calculated the number of polymorphic sites (sites with at least two alleles) and informative sites (sites with multiple alleles found in at least two sequences) for sequences collected up to and including that time. (TIF) [file pcbi.1008537.s002.tif]

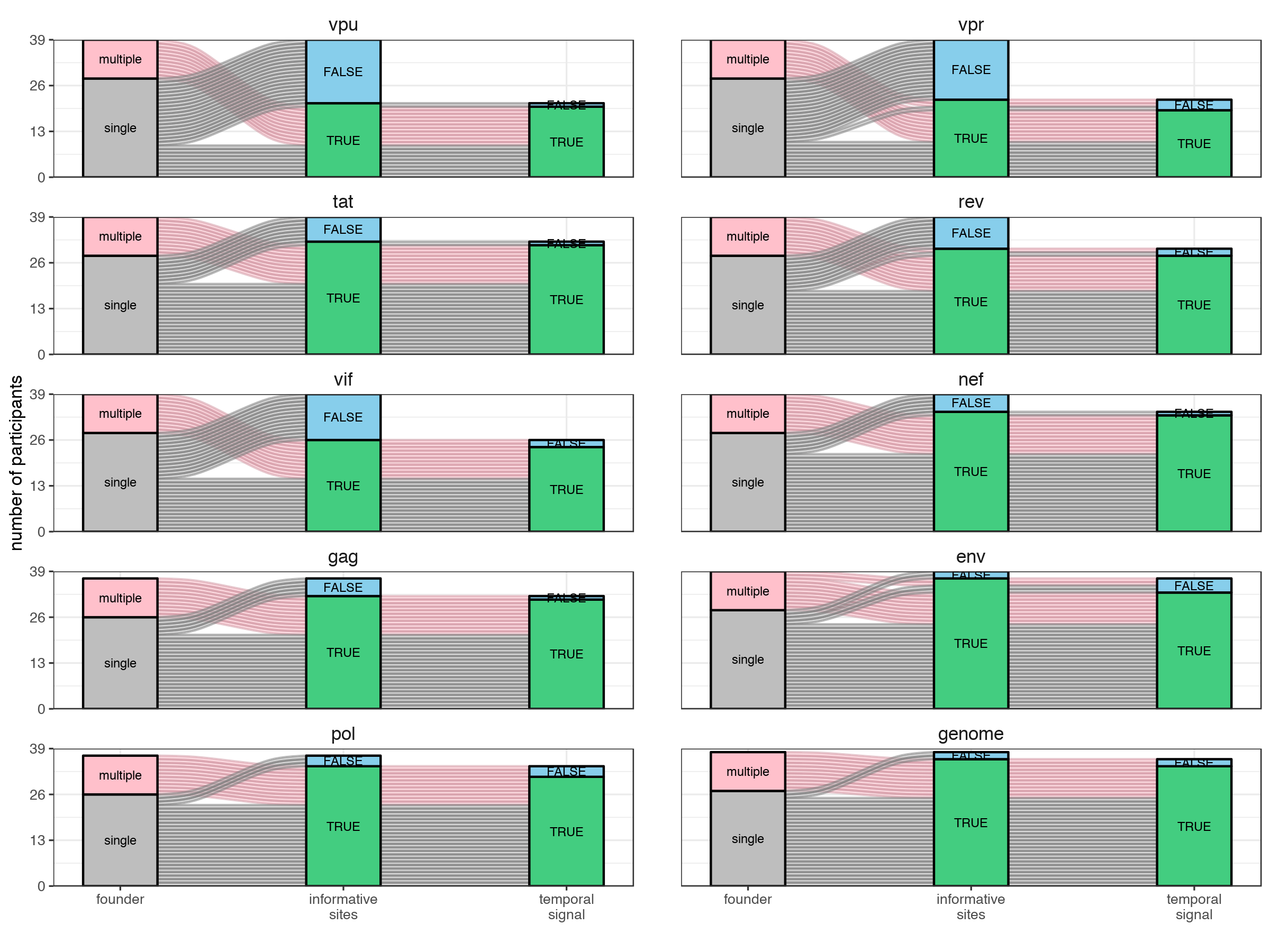

Supplement: S2 Fig — Two participants did not have samples for all three time points for gag and pol and were thus removed for these genes; participant 20368 was removed from the NFL genome analysis due to half genomes only being available. The category ‘informative sites’ refers to participants whose sequences had at least one informative site; these were then tested for significant temporal signal. Only those with at least one informative site and temporal signal were analyzed in BEAST. (TIF) [file pcbi.1008537.s003.tif]

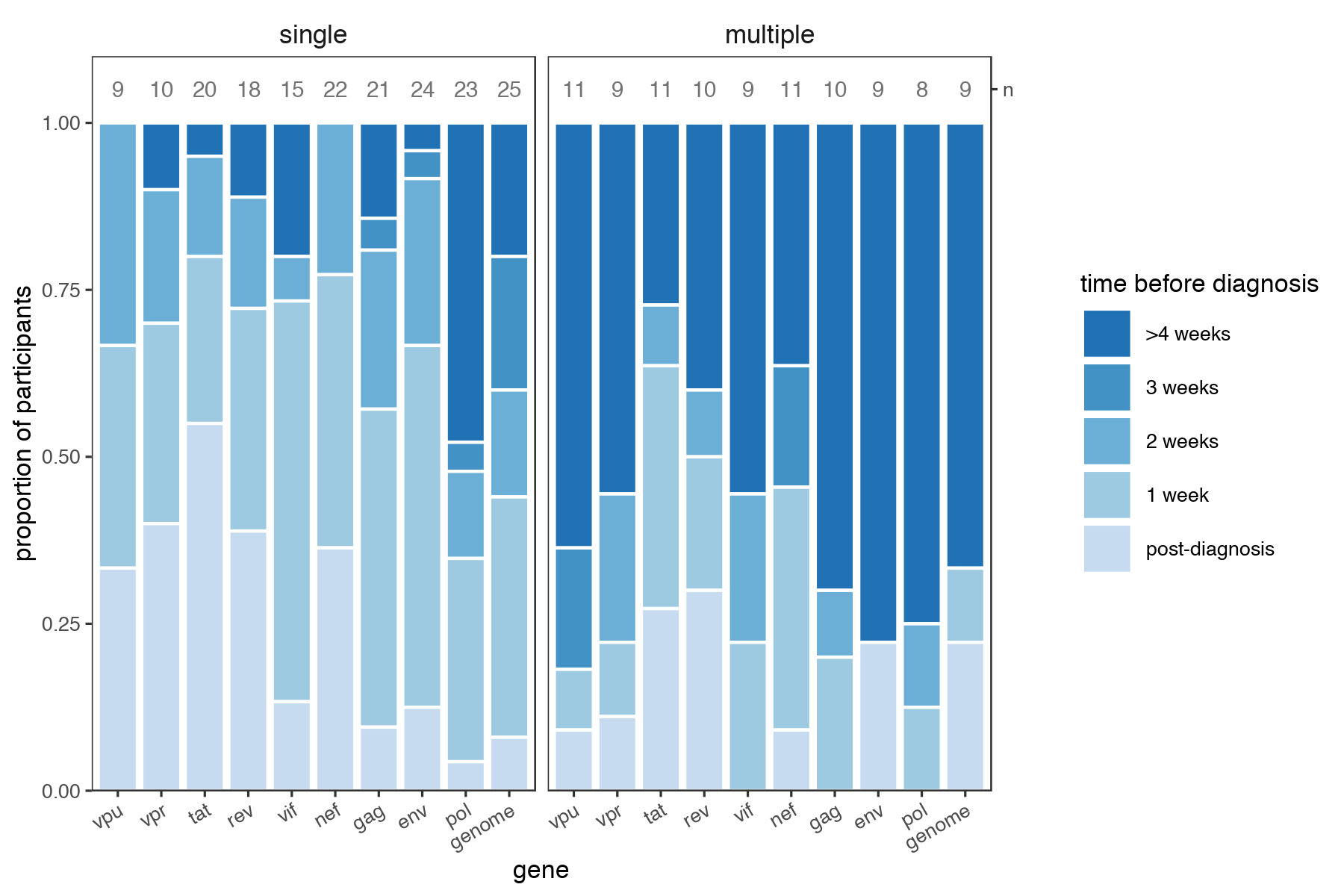

Supplement: S3 Fig — (TIF) [file pcbi.1008537.s004.tif]

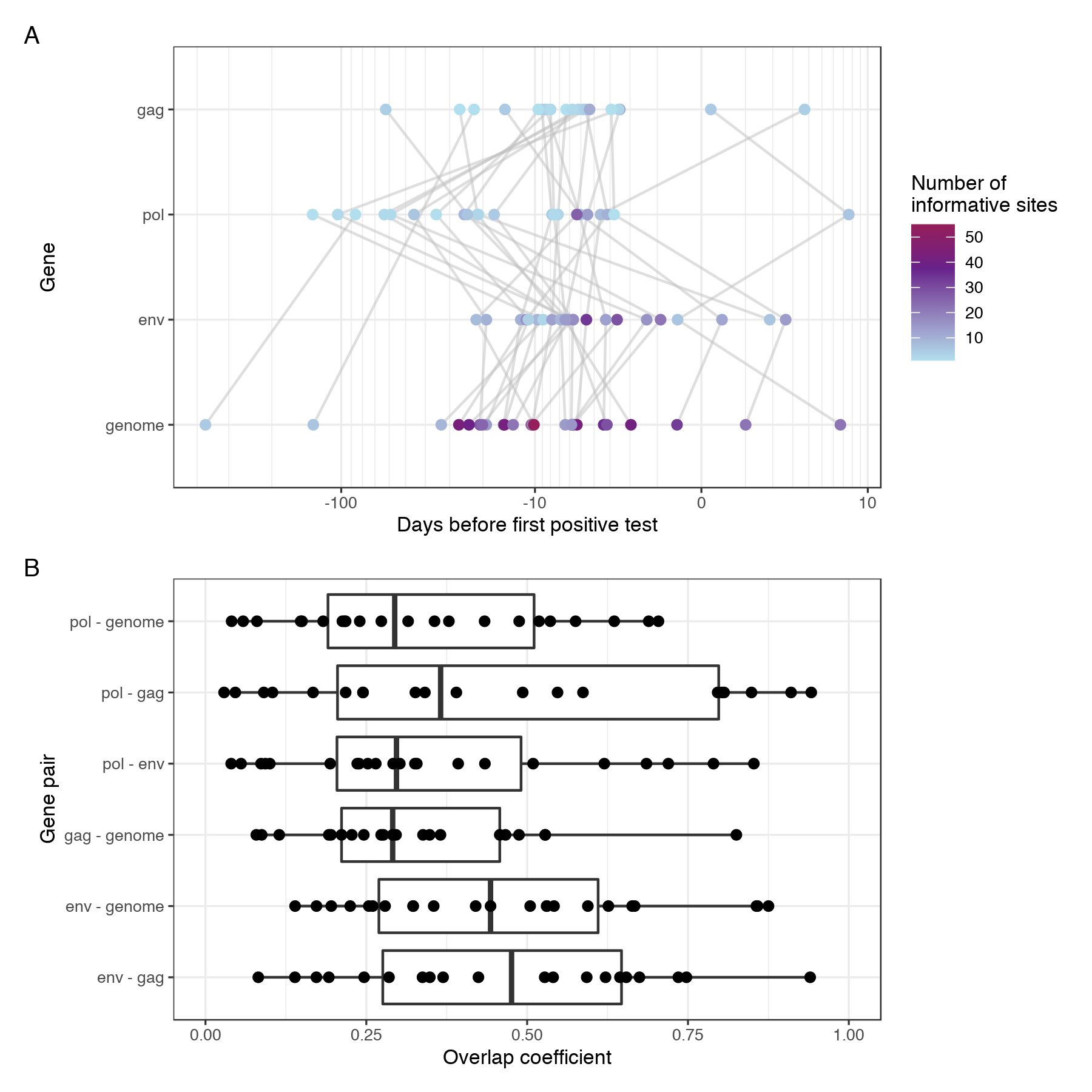

Supplement: S4 Fig — A) Point estimates of the date of infection for participants. The color of the points shows the number of informative sites in the within-host dataset for that participant and gene. Lines link gene and genome results for each participant. B) Boxplot of the overlap coefficient for posterior distributions between pairs of genes for each participant. The coefficient is defined between 0 and 1; 0 implies the curves are non-overlapping, and 1 complete overlap. (TIF) [file pcbi.1008537.s005.tif]

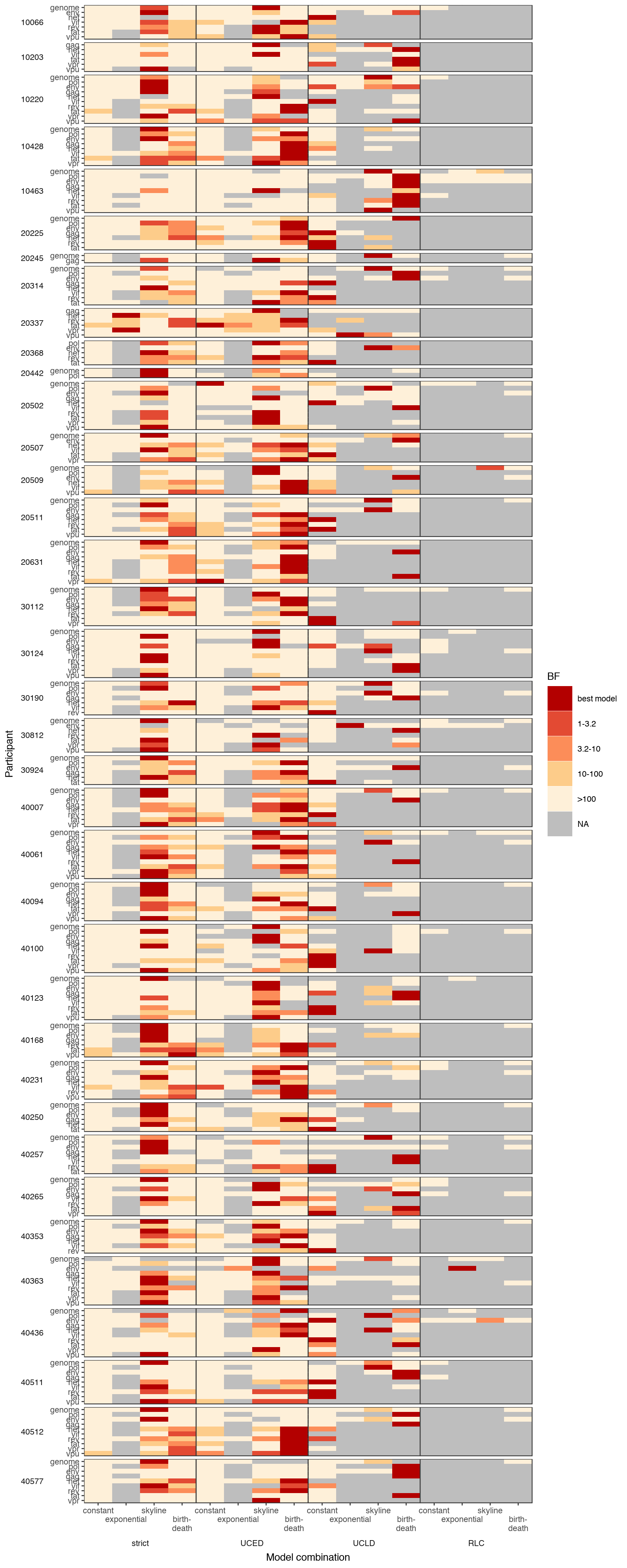

Supplement: S5 Fig — The best-fitting model is shown in red. Shades of orange and yellow give the strength of evidence for the best-fitting model relative to the other model combinations fitted, that is, the darker the color, the smaller the improvement by the best-fitting model. Model combinations shown in gray could not be fitted. (TIF) [file pcbi.1008537.s006.tif]

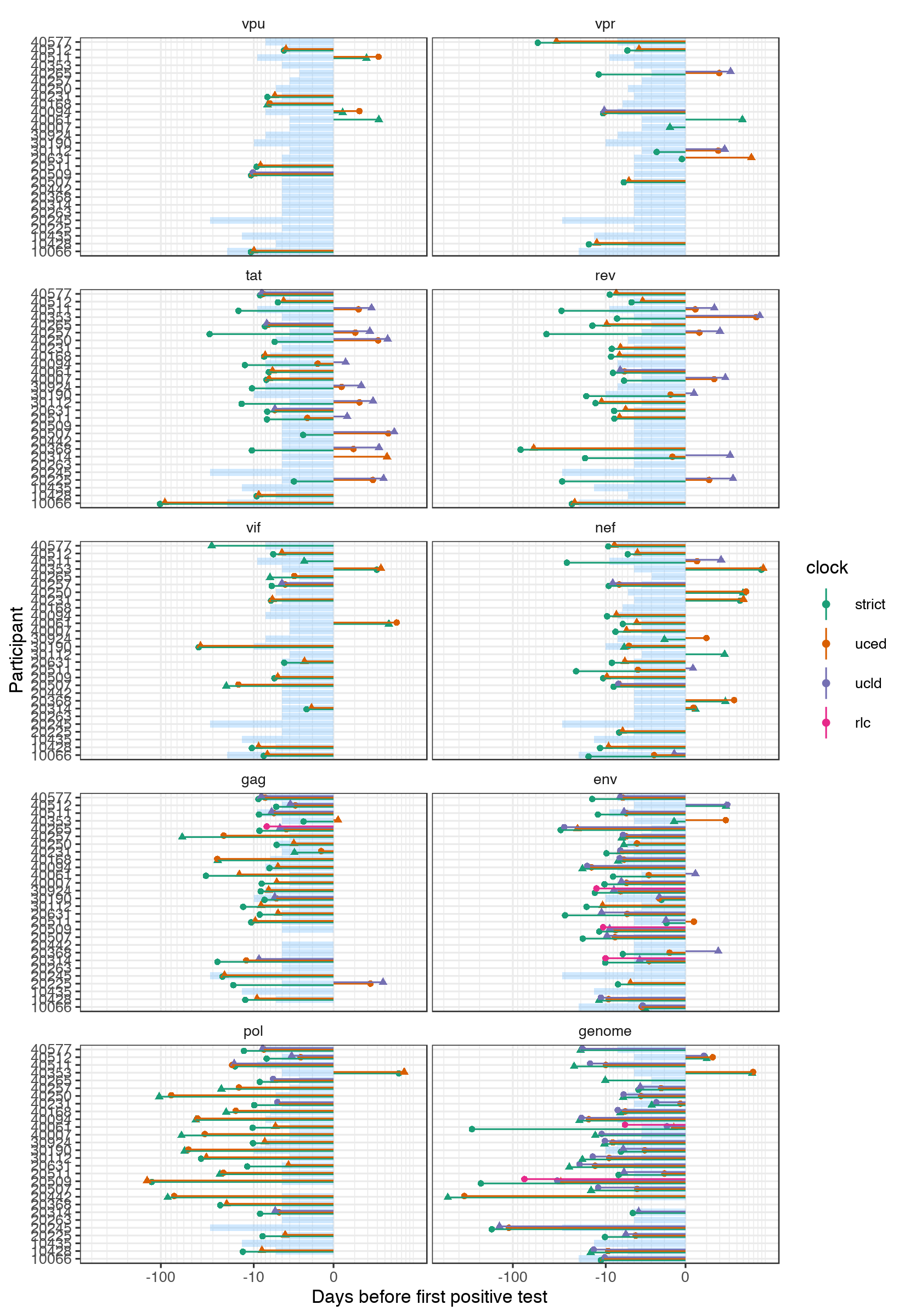

Supplement: S6 Fig — For each participant, the best-fitting estimate from BEAST is marked by a triangle, with a circle showing the result from the other clock models under the same population model. The shaded blue area corresponded to the interval between the last negative and first positive HIV-1 RNA test; where the blue bar is missing, the sequences were unavailable for that participant. (TIF) [file pcbi.1008537.s007.tif]

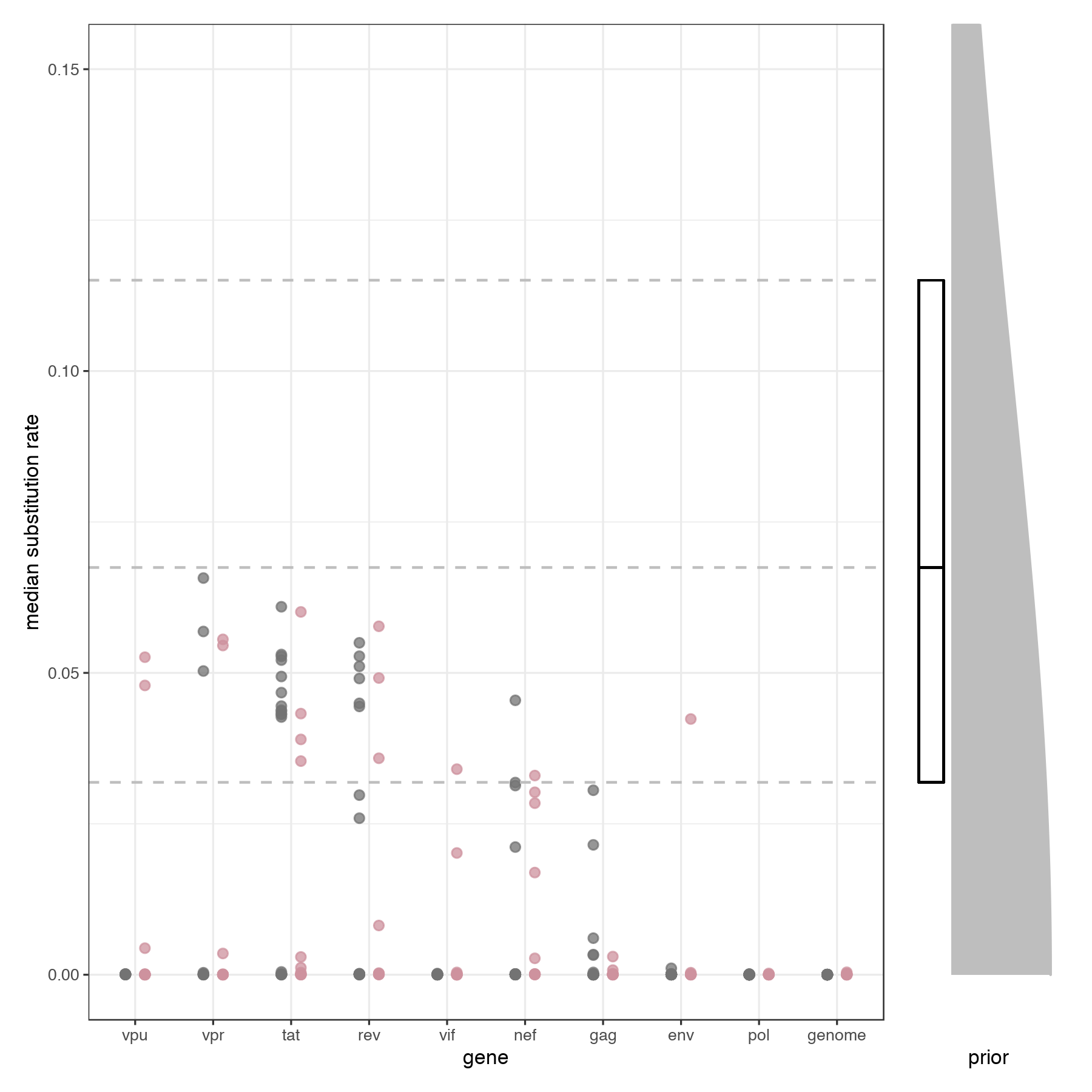

Supplement: S7 Fig — The estimated substitution rates for each participant and gene (left) are plotted alongside the prior distribution (right). Horizontal dashed lines show the median and IQR of the prior. (TIF) [file pcbi.1008537.s008.tif]
