# Supplementary material for: Factors influencing estimates of HIV-1 infection timing using BEAST
Source: PLoS Comput Biol. 2021 Feb 1;17(2):e1008537. doi: 10.1371/journal.pcbi.1008537 (PMC7877758; doi:10.1371/journal.pcbi.1008537)
Supplement: Attachment — Submitted filename: Dearlove_PLoSCompBio_ResponseToReviewers.5aug20.pdf [file pcbi.1008537.s009.pdf]

August 4, 2020

Drs. Kouyos & De Boer,
Editors, PLOS Computational Biology

Please find enclosed our revised manuscript. We thank the reviewers for their thorough evaluation of our manuscript and followed their suggestions to revise our manuscript.

Among the main modifications, we reorganized the text and revised all the figures for greater legibility. We also edited the text to more clearly distinguish infection and diagnosis.

We also slightly edited the title from "Factors influencing estimates of HIV-1 acute infection timing using BEAST" to "Factors influencing estimates of HIV-1 acute infection timing using BEAST".

Please see below for our responses (in blue) to the comments from the reviewers (in black).

We thank you in advance for considering our manuscript for publication in PLOS Computational Biology. Please do not hesitate to contact us if you require further information.

Sincerely,

Morgane Rolland,
On behalf of all authors

Reviewer #1:

Dearlove et al use serially-sampled viral genome sequences from a group of recently-infected HIV patients whose plausible seroconversion dates are known with some precision. They analyse these to determine the level of genetic diversity present in samples at different time points. They then run BEAST with a wide variety of models on each within-host dataset and use model selection to recommend optimal settings. This is a useful study, taking advantage of an unusually comprehensive dataset to make recommendations for general practice. The analyses presented are sensible and well-conducted. I do, however, have a number of substantial concerns. Some concern choices of what to emphasise in the results, others undiscussed limitations.

The manuscript does not actually mention what value it uses as the estimated date of infection (and nor does the accepted Rolland et al. paper). I am assuming, in the absence of a description of any other method, that this is simply the time of the tree root.
But it should be noted that this is not necessarily the same thing, and that results that the authors regard as implausible ("infection dates" after diagnosis and differing "infection dates" for multiple founder strains) are in fact perfectly reasonable if the common ancestor of a sample existed some time after the point of infection. TMRCA estimates that are more recent than would be expected from epidemiological data may not really be a problem. This issue certainly should appear in the discussion.
We thank the reviewer for highlighting this omission. We have updated the Introduction, Methods and Results to make this clearer:
Introduction: 'We previously analyzed env sequences to estimate the eclipse phase using the time to most recent common ancestor (TMRCA) of the within-host phylogeny of sequences from the first six months of infection [8]. Here, we used the same methodology to compare estimates of the date of infection for the same study participants using individual genes and NFL genomes.'
Methods: 'The date of infection was estimated as the root height of the phylogeny; unless otherwise stated, we used the posterior median for point estimates alongside the 95% highest posterior density interval [47]."
Results: 'We took the median time to most recent common ancestor (TMRCA) of the best fitting model (selected using the highest marginal likelihood estimated by stepping-stone sampling) as a point estimate for the date of infection (Figure 3).'
For the second point, the reviewer is correct that the results that we 'regard as implausible ("infection dates" after diagnosis and differing "infection dates" for multiple founder strains)' are methodologically sound and correct from a technical standpoint. However, such results are biologically implausible given the information we have on the participants (multiple consecutive negative tests, specific date of diagnosis. and VL kinetics in acute infection). We have used implausible to describe the discrepancy between technical accuracy vs. biological knowledge.
'We found two scenarios where estimated dates were implausible: estimates that occurred after the first positive test, and estimates that fell many weeks or months prior to the last negative test. Both are problematic for accurately dating when infection took place, but neither are a methodological issue per se. The date of infection was estimated by the time to most recent common ancestor in the tree. For infections dated after diagnosis, this suggests that either the sampled sequences have an ancestor more recent than the one that founded the infection, or, possibly, that the clock rate is being overestimated. In both cases, increasing the sequence sample size by adding more sequences at each time point, or more time points, would help better capture the extant lineages and improve estimates. Over-estimates of the time to most recent common ancestor were most commonly seen for participants infected with multiple founders. The phylogenies for these participants had long deep branches, reflecting evolution both in the current

host, and in ancestral branches in the previous host prior to transmission. The longer this previous host has been infected, the more mutations their viral population will have accumulated and the more diverse the variants they will likely pass on. As a way to try and overcome this, we attempted to distinguish founder variants, and re-run the analyses for each variant separately. This improved estimates substantially (Figure 7), though these were still often unrealistic compared to the known dates of the last negative and first positive HIV-1 RNA tests. For four participants infected with multiple founders at least one subpopulation estimate gave dates after diagnosis, most likely due to the small sample size, as discussed above, whilst four others had estimates more than a month before their last negative test. In the cases where phylogenetic analyses could be conducted on two founder subpopulations, participants had at least one plausible estimate for the date of infection.'

The manuscript also places too much emphasis on point estimates. It's not really surprising or informative to learn that that posterior medians for the time of infection are not consistent between genes or founder strains. More interesting would be how much overlap there is between the relevant posterior densities.

We agree that there was previously an overemphasis on point estimates and we updated Figure 5 to add the posterior densities for each gene to show this overlap. We also added a new S4 Fig, which shows the overlap coefficient between posterior distributions for pairs of genes for each participant. We have updated the text as follows:

'We used the overlap coefficient, which ranges from 0 (no overlap) to 1 (complete overlap), to capture the overlap in posterior distributions between genes for each participant. Coefficients varied widely overall, but tended to show more overlap between posterior distributions for *env* and *gag*, and *env* and genome (S4b Fig).'

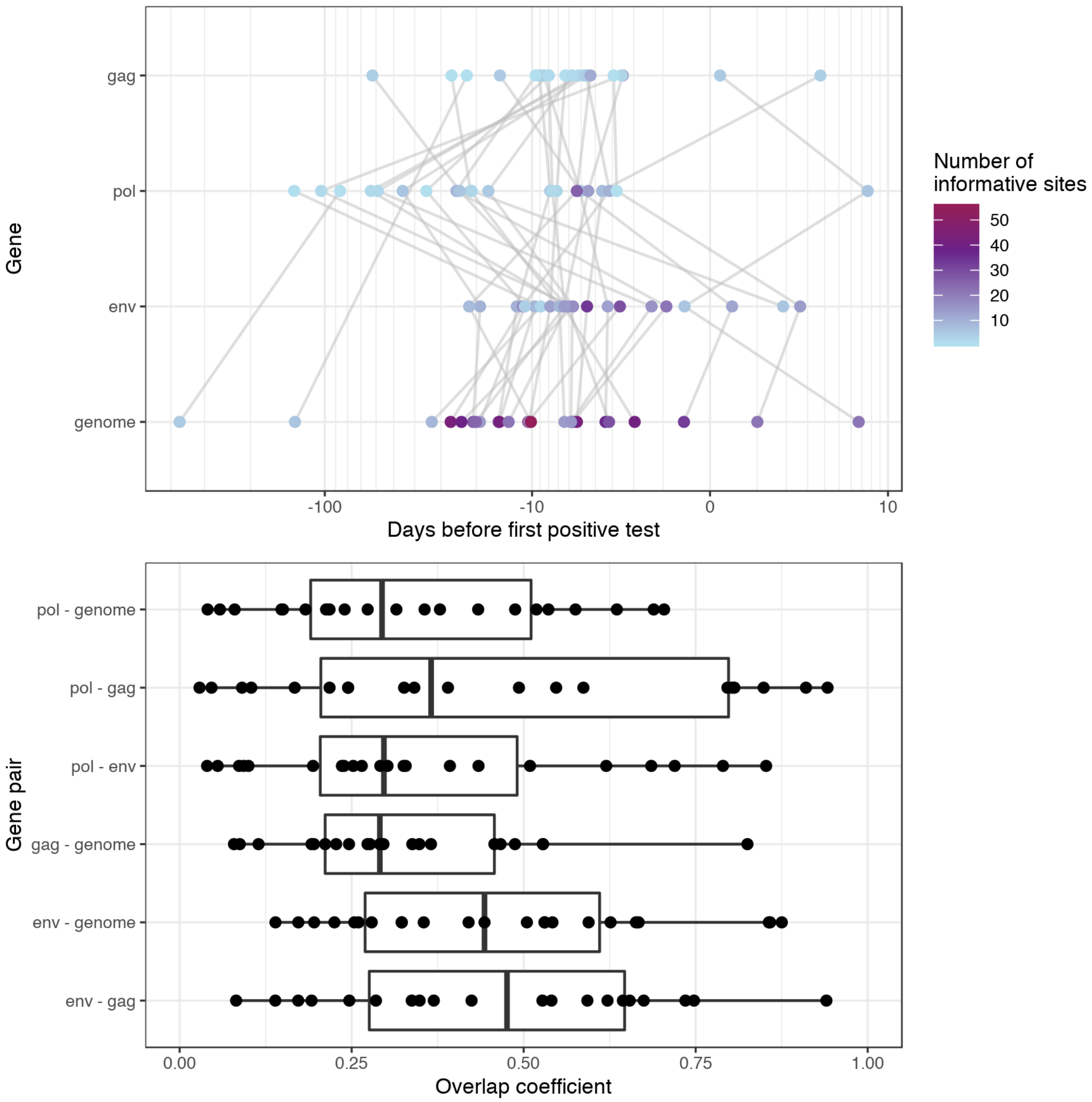

It feels a little unkind to recommend additional BEAST analyses given the potential time and CPU investment involved, but I do feel that some important models are missing and warrant inclusion. Firstly, the BEAST development team would not have recommended use of the original skyline for many years; the first version with a GMRF smoothing prior appeared in 2008. That the skyride and skygrid are appropriate and preferable in almost all circumstances where the skyline would be used seems to have been poorly communicated to the general community of users. I would be interested to see if results using one of them are meaningfully different, and it would make these results reflect current best practice. Secondly, a relaxed uncorrelated Gamma molecular clock was also added to BEAUti some time ago, and this is a more flexible model than even the lognormal as it can also take a shape with a mode of zero (as with the exponential clock) as well as the peaked shape.

We thank the reviewer for raising these points. As they point out, adding 9 new combinations of models (skyride * 5 clock models + uncorrelated Gamma clock * 4 population models) across 9 genes and NFL genome and 39 participants is a large investment of time and would likely have

required extensive modification of the manuscript and figures. We chose to mention these concerns in the discussion:
'Of course, model selection is also dependent on the models fitted; it is also possible none of the models fitted were ideal. This could have been mitigated by including other flexible models, such as the uncorrelated Gamma relaxed clock, or by using the skyride or skygrid population models, which require less user specification than the skyline model we included [33–35].'

The study has access to enviably detailed and precise data on possible time windows for transmission events, but the manuscript makes surprisingly little use of it. For example, how often does the best-fitting model from stepping-stone sampling estimate an infection date within this window? (Or a credible interval which considerably overlaps it?) Does it ever happen that a less well-fitting model gives a more consistent estimate? Which genes perform best in estimating dates that are consistent with the given intervals?
We now show the window between last negative and first positive test for each participant in Figure 5. This window gives us a precise interval as to when the virus became first detectable via RNA test, as the last negative test occurred a median of four days before the first positive test (which we call diagnosis or day 0). However, it would not be correct to use the last negative date as a lower boundary for the time of infection, due to the time it takes for a localized infection to become systemic and for HIV-1 RNA to be detected in the blood. We estimate that this takes 7-14 days, and therefore often falls before the negative test. As such, it would not be logical to expect estimates to fall in the 4-day window between the last negative and first positive test.
The result section was revised as follows:
'Overall, most of the participants had estimates for the dates of infection that fell in the two weeks prior to diagnosis: 79% for *env*, 71% for *gag*, 52% for NFL genomes, and 43% for *pol* (Figure 3, S1 Table, S3 Fig).'

Finally, while I accept that within-host HIV recombination is a very difficult nut to crack and that it is reasonable to ignore it here, a brief discussion of the problem and how it might have affected the results is warranted.
We agree that recombination is a significant concern that we had failed to acknowledge. Yet, the majority of cases correspond to infections with single founders and since sequences are sampled in acute and early infection, there is little diversification. Because sequences are highly similar, even if recombination processes certainly occur, we are not able to detect recombinants in infections with single founders. In contrast, we identified recombinants among the different founder populations in infections with multiple founders. Obvious recombinants were removed from the analyses. While we agree that recombination is a significant issue for these analyses, we think that the small number of sequences was a more important reason behind the poor estimates for infections with multiple founders. We revised the text in the Discussion as follows:
'Besides the small number of sequences per participant (n = 30), recombination across sequences is also likely to negatively impact estimates. The combination of peak viral load and depletion of target CD4 cells that happens in acute infection means that recombination is likely frequent. While it was not an issue for the homogeneous infections with single founders, we found evidence of recombination across the different populations in infections with multiple founders. To alleviate these concerns, we recommend increasing the sequencing depth at each time point to obtain a sufficiently large dataset to capture and analyze each founder subpopulation separately.'

Minor points

L86: I do not really care for the use of “genome” as a shortening of “whole genome”, even if it is explained here. I have to remind myself what is intended every time I see it.
We have replaced genome by near full-length (NFL) genome as our PCR strategy amplifies near full length genomes, i.e. we do not obtain amplicons with both LTRs to limit the risk of recombination.

L167: How was “significant slope” defined?
We tested the slope coefficient of the root to tip regression using a one-tailed t-test. We edited the methods to make this clearer:
‘Temporal signal was assessed following Temp-Est [12]. We first used the rtt function in the ape package in R to root the phylogeny according to the maximum correlation between tip sampling time and distance to root. We then fitted a linear regression of the tip sampling times against the root distances to estimate the mutation rate [50]. Only data sets with a significant positive regression slope (that is, the null hypothesis of $\beta_1 = 0$ was rejected at the 5% level in favor of the alternative, $\beta_1 > 0$ as tested with a one-tailed t-test), were analyzed in BEAST.’

L191: The authors acknowledge that there was little difference between some of the models, could they not use Bayes factors to quantify this?
We have now included a plot of the Bayes Factors in S4 Fig, showing the evidence in support of the best-fitting model compared to all other models that were fitted for that participant and gene. The text now reads:

`The best fitting model combination, as chosen by the highest marginal likelihood estimated using stepping-stone sampling [15], varied across both participants and genes (Figure 5). For no single participant did all 16 models run and converge. The random local clock (RLC) was particularly poor, only fitted for *env*, *pol*, *gag* and the genome. Even so, it was never fitted with the exponential population model, and in all but one case there was substantial evidence (Bayes factor>3.2 [16]) towards rejecting the RLC in favor of the best fitting model (S5 Fig). The exception was participant 20509, with a BF of 2.1 in favor of the best-fitting model (UCLD-skyline) relative to the second best fitting (RLC-skyline) model.

For the NFL genome, the most frequently selected population model was the skyline, selected as the best-fitting for all but one of the participants, in combination with the strict clock for 16 participants, the uncorrelated lognormal relaxed clock (UCLD) for 5 participants, and the uncorrelated exponential relaxed clock (UCED) for 3 participants. The remaining participant was fitted with the UCLD clock and birth-death population model. All participants had at least one model combination including a skyline in the top three ranked models, with 14 out of 25 participants having the skyline population model for all of the top three models selected. In addition to participant 20509 mentioned above, there were four other participants with little evidence separating the top two model combinations, with the top two models both involving the skyline: for participants 20314 and 30190, the UCLD was slightly favored over the strict clock (BF= 2 and 1.2 respectively), for participant 10066 the UCED skyline was slightly favored over the strict clock (BF=1.8), and for participant 40007 the strict clock was slightly favored over the UCLD relaxed clock (BF=1.2) (S5 Fig)’

L199: Not really a guaranteed overestimate, surely, as you do not know the truth.
We agree that this language was confusing, and have now clarified this statement: ‘…we found that the strict clock tended to estimate a longer time between infection and diagnosis…’

L200: When the strict clock was selected as the best model, did it still give earlier estimates than the others?
It did. We now give the differences between the strict clock and other clock models regardless of whether it was the best fitting clock. The updated paragraph reads:
'When considering the best fitting population model, we found that the strict clock tended to estimate a longer time between infection and diagnosis compared to other clock models when in combination with the best population model (S6 Fig). For NFL genomes, the strict clock was estimated to be a median of 2.7 (IQR: 1-12) days prior to estimates using other clocks; strict clock estimates also tended to be more consistent with UCLD relaxed clock (median difference: 1.2, IQR: 0.41-4.8) than the UCED relaxed clock (median difference: 5.7, IQR: 2.2-13). Similar patterns were seen across the other genes, with the strict clock giving estimates 2.5 (IQR: 1.8-5) days prior to other clock models for *gag*, 3.4 (IQR: 0.93-7.2) for *env*, and 3.4 (IQR: 0.93-7.2) for *pol*.'

L220: The pol observation seems quite striking, and I would be interested to see possible explanations in the discussion.
It remains unclear to us why the date of infection tended to be overestimated when using pol data. We have added this to the discussion:
'It remains unclear why the *pol* results tended to differ. Previous work showed that *env* has an elevated evolutionary rate compared to *gag* and *pol*, and that selection acts differently upon the 3' and 5' halves of the genome [18]. The NFL genome results likely reflect the average of these effects between genes, however, given that the estimates from gag were similar to those from *env*, these factors alone do not explain the discrepancy with *pol*.'

L272: The number of datasets mentioned here do not sum to 11, nor is the change from 7 (L267) explained.
We have edited these sentences to hopefully make clearer the breakdown of the number of subpopulations identified for each of the eleven participants. The number of sequences linked to each founder population is also reported in Figure 7. The text was revised as follows:
'Five of eleven infections with multiple founders were split into two founder variants and four participants showed three founder lineages; sequences from participants 30812 and 40363 were split into six and five subpopulations respectively. In some participants, the proportion of sequences attributed to each founder were fairly even (e.g. 10203, 10220, 40123), whereas in other participants one variant dominated with >75% majority (e.g. 10463, 20502, 40100, 40436) (Figure 7).'
In the paragraph above in the Results section, we clarified the number of datasets (n = 7) used in the previously published analysis as follows:
'Of the eleven participants infected with multiple founders, seven showed two variants with sufficient phylogenetic signal for estimates of the date of infection to be compared directly. In five participants, the two subpopulations gave estimates of the date of infection that were more than 24 days apart [8]. To see if the increased information in longer sequences could help better resolve estimates, we repeated the analysis using NFL genome sequences, which are over three times longer than *env*.'

L280: Which BEAST model was used here?
The full model selection was run on each of the identified founding variants, with the best-fitting model results reported. We edited this sentence to clarify:

‘Comparing the best-fitting model estimate from each subpopulation versus all sequences combined, there was a median improvement of 228 days (IQR: 86-404), though the range was still wider than the previous env analysis (IQR: 20 days pre-diagnosis to 0.051 days post-diagnosis).’

L451: It should be noted that improper priors should not be used in combination with stepping-stone sampling. Were all those replaced with proper ones?
Yes, priors had been replaced and proper priors were used for the model selection.

L453: Including the birth-death model at all is a questionable choice. It was, after all, developed as a between-host epidemiological model. I can see how it might be interpreted as a within-host demographic model, but there is also a problem of correlation between the three parameters (see Stadler et al, PNAS, 2013 and in particular the appendix). One of the priors needs to be informative for this analysis to give useful results, and this would have to be carefully chosen if moving the entire framework to a model of a within-host population. This did not happen here.
We agree with the reviewer and, in retrospect, consider that we should have omitted the birth death model from our analyses. While the birth death model could offer a useful framework for within-host scenarios (to account for the stochasticity associated with the transition from a localized infection to a systemic ione), we did not perform any work to adapt this model here. Because this paper is linked to an initial study presenting results on the *env* gene (which included the birth death model), we decided to present comparable results in this manuscript and described the issues mentioned by the reviewer in the Discussion. The text has been edited as follows:
‘Both the birth-death and coalescent models employed here have some vulnerabilities. The birth-death model is a forwards-in-time model designed for the speciation and extinction of lineages at the population level, and has traditionally been applied to between-host epidemiological models of virus dynamics, rather than within-host. While the birth-death model could potentially offer a useful framework for the transition from a localized infection to a systemic one, we did not evaluate this here. It was also recently reported that a multitude of alternative birth–death models are equally likely to explain a given time-calibrated phylogeny [36], and results are prone to bias if the sampling process is misspecified [37]. It has been suggested that one way to avoid potential problems is to always consider the full posterior distribution, rather than putting emphasis on point estimates [38]. However, the full posterior distribution is highly dimensional, which may not be easy to incorporate into clinical trial inference. The deterministic coalescent models are more computationally efficient than birth-death models, being a retrospective look backwards-in-time from the tips, but can struggle to deal with demographic stochasticity [39], which we might expect early in the establishment of infection.’

L463-466: There is a repeated sentence here.
The surplus sentence has been removed.

Most of the figures need enlargement of the label text.
We thank the reviewer for highlighting this and have increased the font size accordingly.

Reviewer #2:

I was excited to read this paper based on the abstract. But, despite the obviously massive amount of work that went into this paper, I think that the analysis does not make sense and is misleading. I think that the authors need to re-evaluate what their objectives are and re-think what analysis best supports that objective. After reading the paper several basic things are not clear to me. The stated objective seems to be to provide some guidance on how to use multi-sample data (e.g. clones or NGS) to estimate (unknown) infection times, but the analysis seems to be nearly exclusively focused on poorly executed and misleading phylogenetic model selection. I'm also unclear on some basics like how the samples were used, were all the time samples combined into a single tree (a single tree figure would have resoled this). If that is the cases, the authors are analyzing a situation that basically never occurs "in the field" (almost none of the many HIV sequences in the referenced will have multiple clone/NGS from multiple time points); if each time point was considered separately, then the results were not reported by their time strata. Some of the supplemental also seems to suggest that the analysis was strange (env seems to have one of the lower median estimated evolutionary rates—the authors also use substation rate when they mean evolutionary rate). The authors say that they expect evolutionary rates that are 100 fold lower than the median of the priors. This makes no sense
We thank the reviewer for his thorough review and tried to provide clarifications. As understood by the reviewer, all the time samples were combined into a single tree. There is one tree per gene and per participant (390 trees). We disagree that a scenario with multiple time points sampled from a given individual never occurs in the field as it is a common practice for researchers studying acute and early HIV-1 infection. Multiple time points will be sequenced in the on-going phase III efficacy trials that test VRC01 as prevention (AMP), thus, we consider that the conditions we studied are relevant to current research, including a critical randomized double-blind efficacy trial. We have corrected the substitution rate issue and provide more details regarding the question on the priors in the general comments below.

General comments

It's not at all clear how multiple infection was determined. The reference is to a paper in press and no other mention is made of how this was done or than to say that alignment, phylogeny, and some kind of graph theoretic method was involved.
We used both visual inspection (of sequence alignments, highlighter plots and phylogenetic trees), and quantitative measures (maximum pairwise diversity, ratio of private to shared mutations and the principal eigenvalue of the modified graph Laplacian) with the genome sequences to identify single versus multiple founder infections – this is a standard approach. The article we referenced (citation 8 that the reviewer also mentions below) is now freely available online: https://journals.plos.org/plospathogens/article?id=10.1371/journal.ppat.1008179. This paragraph now reads:
'Infections were classified into single or multiple founders using the genome sequences across all time points and the current standard approach: visual inspection of sequence alignments, Highlighter plots and phylogenetic trees, and quantitative measures of intra-host diversity including the maximum pairwise diversity and ratio of shared to private mutations [8,10]. Additionally, we used a relatively new metric, the principal eigenvalue of the modified graph Laplacian (MGL), which summarizes the diversification patterns of phylogenetic trees and has been shown to positively correlate with the multiplicity of infection [8,11].'

Figure 2 shows results using methods from citation 10 I think, but citation 8 still seems to be the main citation here (which I cannot read because it's not public). I'm also not clear where the time dimension went in this figure. The samples came from 4 to 170 days from infection but this does not seem to be reference in the figure (are these just the first sample?).
Figure 2 shows the principal eigenvalue of the modified graph Laplacian (MGL). The methods underlying this analysis are described in citation 10 in the original submission, which is now renumbered as citation 11. This article was published on November 4th, 2019, under open access at: https://academic.oup.com/ve/article/5/2/vez044/5611339.
A similar analysis was presented for just the *env* in citation 8. This article was associated with the manuscript as supporting information at the time of submission, and has since been published with open access in PLoS Pathogens:
https://journals.plos.org/plospathogens/article?id=10.1371/journal.ppat.1008179).
In short, the principal eigenvalue of the MGL is a summary statistic derived from the tree that has previously been shown by Lewitus and Rolland (citation 11/(old 10)) to distinguish participants infected with single vs. multiple founders. Trees were reconstructed using the sequences from all three time points in a single analysis, and therefore we have only one principle eigenvalue per participant per gene.

In general, it's not clear why the results are not presented as a function of time from infection. The strength of this study is that that time is known.
The results are presented based on the time from diagnosis based on a sensitive RNA test. Though we have tight bounds on the time of diagnosis due to the frequent testing of this cohort, with a median of four days between the last negative and first positive test, the time of diagnosis is not the time of infection. The actual time of infection remains unknown and is our parameter of interest. Indeed, as mentioned above, due to the short period between last negative and first positive, we would expect the date of infection to be prior to this interval due to the time it takes for HIV-1 RNA to be detectable in the blood.
As suggested by Reviewer 1, we now also show the window of detection between the last negative and first positive tests in Figure 5 for reference.

I don't understand the point behind the "best-fitting model combination" and I don't think that the technical execution makes sense. I'll address the latter point first. Even if we assume that the stone stepping estimator is unbiased and perfect, model selection by marginal likelihoods does not make sense in this case. The marginal likelihood will be highly dependent on the choice of model priors (the ML integrates the product of the likelihood and the prior over the parameter space). Given that some of the model formulations have 10s of parameters, and include ones that have no real principled prior defaults, it's almost impossible that the authors have set up a situation where every single prior was truly evaluated for what the most reasonable distribution should be for these datasets. And, in the unlikely situation where they did, those values were not reported in full. I think that it's more likely that they used more-or-less the default priors in BEAST (for unreported values). In this case, this analysis tells us what the default priors of BEAST think about this data. Further, some of the reported values of the priors don't make sense: the constant population size had a lognormal prior (logmean=0 sdmean=1), which has an expectation of about 1.68, while the exponential population size was uniform over 1 and 1 google. The marginal likelihood calculations therefore assume that the likelihood when the population is 1 billion billion billion is as important as when the population size is 100. To extent the critique, if we told two competent HIV phylogeneticists to do this analysis and to carefully think about the priors, we would get two very different sets of "best" models not because either were wrong, but because the outcome is highly

dependent on “reasonable guesses” (e.g. what is a reasonable prior on the exponential growth rate of an ideal population for these data?). This whole analysis does not make sense to me and I believe is highly misleading.

I also find it confusing that the authors seem to want to talk about which models are the best at recovering the known times of infection, but they use instead a (poor in my opinion) model fit statistic. The authors logic is not really stated so I can’t really say, but I suspect that they used marginal likelihoods because they naturally penalize overfitting by naturally spreading out the integral over a larger parameter space in more complex models. However, the whole phylogenetic model is just a nuisance parameter if the only goal is to recover the infection time. If there was a single model that had the lowest bias in the estimated of the infection time but that was only partially identifiable or had a bad marginal likelihood, would that matter? The authors are not trying to do biology but rather to determine the statistical properties of an estimator.
We believe there was some confusion over what our goal was because it had not been clear to the reviewer that we do not know the time of infection. As mentioned above, the interval between a negative test and a positive one does not provide the time of infection, and this is what we aimed to obtain when identifying the best fitting model.
As stated in the methods (section: BEAST model selection), we used stepping-stone sampling to estimate the marginal likelihoods. This has been a standard method for model selection with BEAST analyses since first published by Baele et al (2012; 2013), though has arguably been underused because of the computational time it takes to run both the BEAST analysis and additional marginal likelihood estimation for every model of interest. In our analysis, our aims were two-fold: first, to estimate the time of infection for each participant for each gene and the genome and compare estimates. Since it was unclear which demographic model would best capture the within-host dynamics of early infection, we used model selection to identify the best fitting model from 16 different combinations, as is the standard approach. A secondary follow up to this was to identify whether there were patterns in the models selected across participants; it would have been a valuable result for future work had all of the participants been best fitted by the same model combination (or, at least, a more limited set), as this would save computational time. However, this was not the case. To better reflect these aims, we have reordered the results section, first presenting the results of the best fitting model, and then moving to talk about the patterns in the model selection across participants.
We address the concerns of the reviewer regarding the choice of priors in further responses below in response to Reviewer 3. In brief, we chose to use priors that arguably ‘don’t make sense’ to be able to make sure that the results we obtained were not dictated by the priors. Our main concern with this dataset was that we were at the lower limit in terms of information content for these datasets given that a majority of sequences were sampled in the first month after diagnosis. This is especially true for the short genes in the HIV-1 genome. By choosing wide priors, we were able to identify that the output from the short genes only reflected the priors and should be discounted, while for the longer genes and NFL genomes, our results were driven by the data. Had we used priors that ‘made sense’, our results with the short genes would have looked reasonable despite being methodologically incorrect.

The vertical grid lines in Figure 3 make reading the boxplots hard. This figure also seems to suggest that using more data (whole genome) leads to worse and more variable estimates. It seems that this result alone suggests either the models being used were not sufficient for the

whole genome (i.e. not enough heterogeneity comparted to individual gene models) or that recombination is causing a systemic bias towards longer trees.

We believe that the vertical gridlines are important to show that the plots are not on a linear scale. To help reduce clutter in the plot, we instead removed the horizontal grid lines. We have also adjusted the intensity of the colors, as suggested by Reviewer 3.

For the genome results, the IQR was 20 to 5 days before diagnosis. There were two notable outliers, participants 20442 and 20245, who had estimates more than 100 days prior to diagnosis and skewed the genome results. Without these, the IQR is estimated to be 4-17 days before diagnosis.

This is highlighted in the text as follows:

'Curiously, for trees reconstructed from *pol*, and to some extent NFL genome, sequences seem to overestimate the eclipse phase compared to *gag* and *env* (Figure 4, S4a Fig). Particularly striking were participants 20442 and 20245, with estimates for the date of infection for the NFL genome more than 100 days prior to diagnosis (20442: 371 days prior to diagnosis, 20245: 133 days). These two Kenyan participants quickly controlled viremia after peak viral loads of 6.54 and 6.25 log copies/ml for 20442 and 20245 respectively, and only had 5 (participant 20442) and 6 (participant 20245) informative sites across the three time points for the NFL genome (indeed, only the NFL genome and one other gene had sufficient genetic and temporal signal to be included in the BEAST analysis). The lack of substitutions led to very low evolutionary rate estimates (20442: 6.66× 10-7subs/site/day; 20245: 2.64× 10-6subs/site/day; overall median: 1.45× 10-5subs/site/day), pushing the root of the phylogenies back in time.'

We do not think that the NFL genome results are evidence that the increase in data associated with NFL genomes indicate worse results, rather we think this may be a consequence of the potentially difficult reconciliation of the known imbalance between evolutionary processes in the 5' and 3' parts of the genome. We think that the genome results likely reflected heterogeneity between genes, representing an average across sites evolving at different rates. We added the following description to the discussion of the pol results requested by Reviewer 1:

'It remains unclear why the pol results tended to differ. Previous work has shown that env has an elevated evolutionary rate compared to *gag* and *pol*, and that selection acts differently upon the 3' and 5' halves of the genome [18]. The NFL genome results likely reflect the average of these effects between genes, however, given that the estimates from *gag* were similar to those from *env*, these factors alone do not explain the discrepancy with *pol*.'

Regarding recombination, while we agree that it may affect results for the subset of infections with multiple founders, this is likely not a significant concern for the majority of cases which are infections with single founders. We saw no evidence of recombination in the infections with single founders, though we know it is likely frequent. This was added to the discussion:

'Besides the small number of sequences per participant (n = 30), recombination across sequences is also likely to negatively impact estimates. The combination of peak viral load and depletion of target CD4 cells that happens in acute infection means that recombination is likely frequent. While it was not an issue for the homogeneous infections with single founders, we found evidence of recombination across the different populations in infections with multiple founders. To alleviate these concerns, we recommend increasing the sequencing depth at each time point to obtain a sufficiently large dataset to capture and analyze each founder subpopulation separately.'

Specific comments

(L60) This phrasing is weird, should be made clear that the unknown time is the interval from

infection to diagnosis. Also, “often” is misleading; there is always a lag between infection and diagnosis.
We have removed the ‘often’ as suggested.

(L70) I don’t think of PCR as being a serological assay, but this might be ignorance on my part.
We thank the reviewer for catching this error. We meant ‘besides diagnostic assays’.

(L73) The authors should pick a single term for the time interval between infection and diagnosis and stick to it (in the first 1.5 pages they use both age and date of infection).
We have updated the manuscript to refer to the ‘date of infection’ only.

(L104) Why are there 3 medians reported? Is this the median time for the first, second, and third samples?
Yes, these correspond to the three time points. We have revised the first paragraph of the results to make this clearer:
‘We analyzed HIV-1 genomes from 39 participants sampled longitudinally approximately one week (median: 4 days), one month (median: 32 days) and six months (median: 170 days) after the initial detection of HIV-1 viremia by positive HIV-1 RNA test; this diagnosis occurred a median of four days after the last negative test [8]’

(L106) What does “mainly identified” mean? Subtype A1 was 9/23, do you mean the most common subtype was A1?
We edited the text as the reviewer has suggested.

(L108) The Thai woman mentioned here is omitted in the intro.
This has now been fixed.

(L115) This seem tautological as sequence alignment (and ostensibly number of polymorphic sites) was used to determine multiplicity of infection.
We agree with the reviewer and removed the sentence in question (‘Infections founded by a single virus had fewer polymorphisms (that is, sites with at least two alleles) than infections which were seeded by multiple subpopulations’).

(L126) How do the authors know which patients were actually multiply infected? Do they just mean that each method was internally consistent.
We have clarified above in our response to the first general comment how we identified infections with single vs. multiple founders, specifically by evaluating multiple metrics calculated for NFL genomes.
Line 126 refers to the comparison of the results obtained from NFL genomes vs. the different genes, i.e. ‘Using phylogenies reconstructed in IQ-TREE, we investigated the signal for multiple founder infections using individual genes and compared them to the genome classifications’. Here we investigated whether the phylogenetic trees alone, especially those of the shorter genes, could be used to reliably separate single versus multiple founders using the principal eigenvalue of the modified graph Laplacian (MGL). While these results are not central to our study, they also serve to emphasize that results from the shortest genes may be less reliable.
We have updated this paragraph as follows:
‘The single versus multiple founders classification above was obtained using NFL genomes and we wanted to investigate if one of the methods used, the principle eigenvalue of the modified graph

Laplacian (MGL), could yield similar results across the different genes despite the low number of polymorphic sites in some genes. To define a cut off between the two subsets, we used the median, jump and partition criteria; all three gave consistent results for NFL genomes. Though results were broadly consistent, there was a discrepancy between the three criteria for four borderline cases (participant 20225 in *env*, 40061 in *nef*, 30112 in *tat* and 40112 in *vpu*). This was due to the distribution of the data, with the threshold effectively rounded up to the value of the λ^* at the threshold [11]. We also found discrepancies between genes. For example, participant 10220, who had an infection with multiple founders, was classified with the single founders for *env*, *tat* and *rev*, where the sequences were more homogeneous than in the rest of the genome. Likewise, the infection in participant 40265 corresponded to a single founder but was misclassified as a multiple founder for five of nine genes'

Reviewer #3:

Overall this is a very interesting paper dealing with a very common potential flaw in phylogenetic analysis -- using separate genes that may be more readily available rather than using full genomes that contain more information for meaningful phylogenetic analysis. These kinds of articles are extremely important in particular due to the existing status quo in the field, where the genes with limited information may be used to make conclusions on an epidemiological scale.

The dataset that is being used in this and some preceding papers by the same authors is very unique and useful. Showing the use of computational methods to narrow down the date of suspected infection is a great result that has major potential impact on the healthcare system, helping to control spread of infection in real-time.

However, there are two major concerns about the current version of the manuscript that I mention again later where they occur in text. The first major concern is the fact that per dataset of three sequences per patient and gene/genome the authors ran 16 independent analyses. The amount of analyses on one small dataset is large and this inherently leads to some levels of false positivity (e.g. of models fitting "well" to the data while in fact the fit is not that good). Unfortunately, the authors do not mention how they counteract this or why it is not an issue.

The second major issue is the selection of priors on different parameters. The priors were obviously intended to be as uninformative of the true values as possible (e.g. very flat uniform distributions used for different parameters), however these kinds of distributions are far from uninformative for parameters with extremely low expected values. E.g. in cases when a major part of the posterior distribution lies between 0 and 0.1, whereas the prior sets a weight of 9999.9 on values larger than 0.1. Unsurprisingly, when the amount of information is low, the posterior is very close to the prior.

Overall, I do not think that the authors should redo any analyses in order for this paper to be scientifically useful and a helpful addition to the existing research body. Moreover, rerunning analyses under slightly different assumptions may further exacerbate the multiple testing issue brought up above.

However, at the stage that it is now the manuscript is missing some crucial explanation for justifying the choices made for the analyses. The prior selection is very specific but also extremely generic given the body of knowledge already present on HIV evolution. With the addition of choice justification the manuscript should be ready for publication.

Additionally, I think that it would be very helpful if the authors also published their Beast configuration files so that other researchers could analyse their datasets in a similar manner.

We thank the reviewer for their thoughtful comments. Before getting into the specific points, we wanted to address the reviewers' concerns about the sample size, as there has clearly been a misunderstanding about the data we used. For each participant, we have an average of 10 sequences at each of the three time points, or around 30 sequences for each analysis rather than the three the reviewer mentions. The datasets for analyzing the multiple founder variants were smaller (Figure 6), but we excluded any data sets with five or fewer sequences.
For their last point, we will make example BEAST files available on our website: https://www.hivresearch.org/publication-supplements.

Major comments:

Line 39 and multiple others:
The paper reports IQRs for the posterior values. It would seem more reasonable to (also) report the standard 95% HPDs that most Bayesian analyses report (and in particular Beast analyses) as this covers most of the values of the distribution and can show how precise the estimates are. For example, if the 95% HPD range is extremely wide, the precision of the estimate is low and few conclusions can be drawn from the median value. The reported IQRs are already quite wide so it would be interesting to see the 95% HPDs.
The IQRs we report throughout the paper are intended to give an overall summary across participants, that is, the IQR of the medians estimated per participant. We agree that on a participant level the 95% HPD is much more much useful than a single point estimate, and therefore have changed Figure 5 to show the posterior density across the different genes by participant:

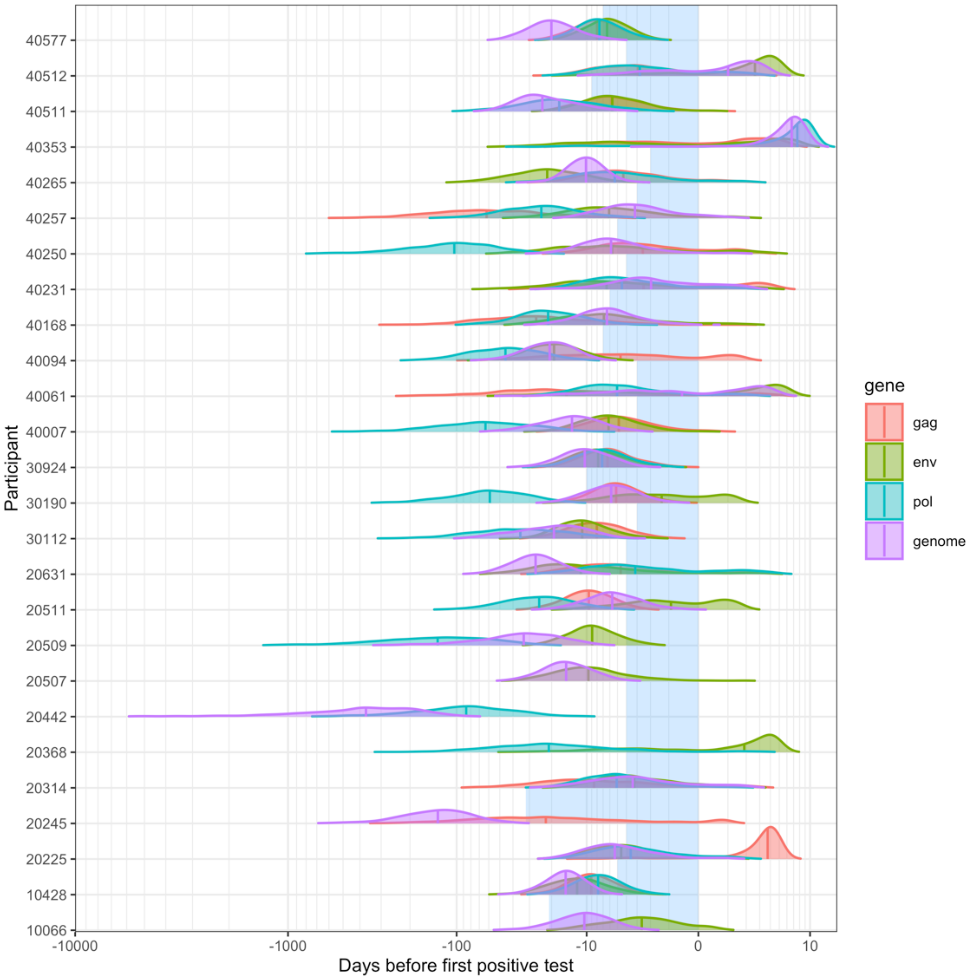

Line 172:
The paper says that there were 9 datasets for a single gene and 25 datasets for full genomes. How is it possible that there were many more full genome sequences (that should include that single gene) than single gene sequences?

The genetic signal was assessed on a gene by gene basis. In general, the shorter genes had little or no evolutionary signal over this time scale (e.g. 60% participants had no informative sites for *vpu*), which also translated into little temporal signal (as assessed by a root to tip regression). When there is no signal for a molecular clock, it is inappropriate to try and estimate a time-scaled tree in BEAST, and therefore datasets for individual genes which lacked shared mutations and/or had no temporal signal were removed from further analyses (as summarized in S2 Fig).

Line 176:
One major concern is that small datasets were re-analysed multiple times setting up different models. While a legitimate criterion was used to determine goodness of fit, I am concerned that the multiple analysis runs have introduced false good fits. Could the authors clarify how this was handled or why it is not an issue?
We agree with the reviewer's concern that our procedure could identify false good fits, given the number of combinations tested using a relatively small dataset (though it is the largest dataset currently available to do this). Because the goal of our study was to compare estimates across genes and models, it was difficult to avoid this issue. We hope that our interpretation of the data shows that we highlight that estimates are sensitive to multiple variables (and not that we could pick a good one). We have tried to emphasize in the manuscript that a small number of sequences, short sequences and limited sequence diversity negatively impacts which models fit (Figure 5) and how well (S5 Fig). While the concern raised by the reviewer is legitimate for our study, we hope that scientists will read our results as showing that one needs to be cautious when making conclusions based on a single gene (sometimes with limited model testing). We added a final sentence to the discussion: 'More broadly, our results emphasize that further studies should be done in parallel across different HIV-1 genes to better understand how the choice of a specific gene may dictate certain conclusions.'

Line 180:
It is not exactly clear how the analyses are set up in Beast. It is my understanding that individual patient data (single gene or genome) was analysed in a single analysis separate from any other patient data. However, there are only 3 sequences per patient, which is incredibly little for reliable parameter inference. How do the authors counteract that? In particular given that the priors on different parameters such as substitution rates are uninformative (in fact, informative of values very different from the expected) and especially since per participant (i.e. per 3 sequences) 16 different model configurations were ran.
As we mentioned above, 30 NFL genomes were analyzed per participant. We revised the first paragraph of the results to make the number of sequences clearer:
'We analyzed HIV-1 genomes from 39 participants sampled longitudinally approximately one week (median: 4 days), one month (median: 32 days) and six months (median: 170 days) after the initial detection of HIV-1 viremia by positive HIV-1 RNA test; this diagnosis occurred a median of four days after the last negative test [8]. Ten HIV-1 genomes were derived via endpoint-dilution from plasma samples for each participant at each time point, resulting in 1,280 total sequences. In East Africa (n=23 women), the infections were most commonly identified as subtype A1 (n=9), followed by subtype C (n=3) and various A1/D recombinants. In Thailand (n=10 men, 5 transgender women and 1 woman), all but two of the infections were CRF01_AE (n=14); the remaining infections were subtype B (male participant) and a B/CRF01_AE recombinant (transgender participant).'

Line 103:
Were the differences in subtype evolution accounted for? E.g. it could be that different subtypes

evolve at very different rates, which may bias the analysis results. In particular, when trying to estimate exact timescales good priors on evolution rates can be crucial for precise estimates.
Here we used the use same clock prior across all analyses, based on analyses we had performed in our prior study which used the *env* gene from the same dataset (Rolland et al. (2020)):
https://journals.plos.org/plospathogens/article?id=10.1371/journal.ppat.1008179
In that study, we had showed that changing the truncated normal prior to a Unif(0,1) yielded similar estimates for both the substitution rate and age of infection with the best fitting population model for env (Supplementary Figure 6 of Rolland et al (2020)), regardless of subtype. These results indicated that it was the data that was driving the posterior distribution. As mentioned in that prior study, the different subtypes found in the cohort together with other differences, such as sex or ethnicity, combined with the relatively small number of participants (n = 39) meant that we could not meaningfully interrogate the question of the HIV-1 subtype.

Line 320:
This is a very true statement, analyses with little data do end up mainly reconstructing the priors. This is the primary reason for using more data and informative priors. This statement raises once more the question of prior selection described in the manuscript as a lot is already known about HIV phylogenetics and the priors selected here were extremely broad.

Line 324:
Here is an example of the prior explicitly putting emphasis on an unrealistic value. The only conclusion that can be drawn from this would be that there is not enough information in the data for successful inference.
Line 452:
The prior choices described here need further justification.
"a uniform prior between 0 and 100,000 for the birth rate" -- This is in fact a prior that puts an incredibly high weight on any value above 1 (99,999 vs 1).
"a uniform prior between 0 and 100 for the rate of sampling through time" -- The sampling proportion and rate in this case are values that are relatively well known for the background population. It would make more sense to set them sensibly based on existing knowledge to not bias the analyses.
"a uniform prior between 1.0 and 1.0 x 10^100 was used for the time the lineage originated" -- in this case as well, the oldest human lineages are from the 20th century at the earliest and the prior puts a lot of weight on values a long time in the past.

We understand why both reviewers 2 and 3 questioned our choice of priors. They are correct but we still think our strategy was right given our objective. Our rationale was that sufficient data will overwhelm a not ideal (or even a poor) choice of prior and will produce reasonable posterior distributions for inference. Although a more informative prior could help improve estimates, it could also cover the fact that there is not enough information in the sequences to drive the signal for the estimate and give misleading results (Garrett and Zeger, 2000, Biometrics). We believe that it was fundamental for our study to determine instances when our sequence data were not informative for parameter estimation. We revised the discussion as follows:
'Most genes had fewer than 30 informative sites across the three sampled time points, with more than 60% of participants having no informative sites found in the shortest genes (*vpu* and *vpr*). This is problematic for parameter-rich phylodynamic analyses, and we found that the lack of signal gave estimates heavily influenced by the prior distribution rather than being biologically plausible (S7 Fig). As an example, we would expect estimates for the evolutionary rate on the order of 10^{-5}

substitutions per site per day (derived from [22]). However, over one third of the point estimates for *vpr*, *tat* and *rev* were more than 100 times higher than this, and reflected the median of the prior, which had a median of 0.067. Substantial prior posterior overlap, with the prior distribution driving the posterior, suggests that the data is having little influence on the results and this non-identifiability can give misleading results [23]. One way to overcome this could be a more informative prior, although it could also serve to exacerbate the problem and obscure that there was not sufficient information to drive the output. We recommend checking the prior posterior overlap to check for identifiability problems, especially for short genes with low genetic diversity [24].'

Line 350:
Could it actually be the estimate of the infection time for the infection source for that patient? I.e. if we are estimating the time of coalescence of the founder variants that all come from a single other source, it would span within-host evolution over two patients -- the sampled patient and the source of infection. Of course, there's a possibility of multiple founders in the source too, but the exact same founders would be extremely unlikely to have been passed on to the sampled patient.
This interpretation is correct. We have expanded the discussion to reiterate this point:
'Over-estimates of the time to most recent common ancestor were most commonly seen for participants infected with multiple founders. The phylogenies for these participants had long deep branches, reflecting evolution both in the current host, and in ancestral branches in the previous host prior to transmission. The longer this previous host has been infected, the more mutations their viral population will have accumulated and the more diverse the variants they will likely pass on. As a way to try and overcome this, we attempted to distinguish founder variants, and re-run the analyses for each variant separately. This improved estimates substantially (Figure 7), though these were still often unrealistic compared to the known dates of the last negative and first positive HIV-1 RNA tests. For four participants infected with multiple founders at least one subpopulation estimate gave dates after diagnosis, most likely due to the small sample size, as discussed above, whilst four others had estimates more than a month before their last negative test. In the cases where phylogenetic analyses could be conducted on two founder subpopulations, all participants had at least one plausible estimate for the date of infection. These results suggest that with more sequence data available, it should be possible to identify with more confidence the estimated date of infection based on the main founder population.'

Minor comments (typos, typesetting errors, etc):
Line 28: The abstract says "coalescence analyses" whereas in fact the paper presents analyses based on both the coalescent and the birth death models.
This has been updated to 'Bayesian phylogenetic analyzes'.

Line 181: The word "were" seems to not match the rest of the sentence structure (maybe it is redundant?). We have now removed the clause referred to in this sentence.

Line 203: Missing a comma after "but when not".
This sentence was also removed.

Line 214: "emcompassing" --> "encompassing".
This was also removed.

Line 271: The authors refer to 5 out of 11 datasets as the majority, which is not the case.
This has now been corrected: 'Five of eleven infections with multiple founders were split into two founder variants and four participants showed three founder lineages; sequences from participants 30812 and 40363 were split into six and five subpopulations respectively.'

Line 361: "straight forward" --> "straightforward". Fixed.

Line 463: The first sentence of the paragraph is repeated twice. This was corrected. Thank you.

line 470: The parameter of the Beta distribution is typeset as a blank square. Fixed.

Figure 1: The grey and the pink are a bit dull and therefore not easy to see.
Figure 2: The figures are too small to be able to read the sample numbers -- the fonts need to be bigger as well as the figures themselves.
This issue was also raised by Reviewer 1, and both figures and font sizes have been adjusted.

Figure 3: The grey and the pink are a bit dull and therefore not easy to see.
We have increased the intensity of the colors.

Supplementary Figure 3: The plots are unreadable due to the small size.
The figure was modified. Thanks for the suggestions.
