# Supplementary material for: Factors influencing estimates of HIV-1 infection timing using BEAST
Source: PLoS Comput Biol. 2021 Feb 1;17(2):e1008537. doi: 10.1371/journal.pcbi.1008537 (PMC7877758; doi:10.1371/journal.pcbi.1008537)
Supplement: Attachment — Submitted filename: 20200928_Dearlove_PLoSCompBio_ResponsetoReviewers.pdf [file pcbi.1008537.s010.pdf]

September 28, 2020

Drs. Kouyos & de Boer,
Editors, PLoS Computational Biology,

Please find enclosed our revised manuscript. Following guidance from the editors, we modified our title from "No selection for HIV-1 mutations in rebound viruses after VRC01 infusion and analytic treatment interruption" to “Neutralizing antibody VRC01 failed to select for HIV-1 mutations upon viral rebound”.

We thank the reviewers for their helpful and constructive feedback and followed their suggestions to revise our manuscript.

Please see below for our point-by-point response (in blue) to the comments from the reviewers (in black).

We thank you in advance for considering our manuscript for publication in PLoS Computational Biology. Please do not hesitate to contact us if you require further information.

Sincerely,

Morgane Rolland,
On behalf of all authors

Reviewer #1:
My concerns have largely been addressed, but I have a few follow-up points:

L104: I did not spot this the first time, but ten sequences from three time points in 39 individuals would be 1170 sequences, not 1280.
We had approximately 10 genome sequences for each participant at each time point (about 30 genomes total), with up to xx sequences per participant. For some participants, ten NFL genomes could not be amplified and pairs of 3' and 5' half genomes were sequenced instead. Additional *env* sequencing was also undertaken for some participants – for example, participant 20263 due to the intriguing low diversity found across the genome. As both reviewers highlighted the discrepancy, we re-wrote the text:

'We analyzed HIV-1 genomes from 39 participants sampled longitudinally at three time points after the initial detection of HIV-1 viremia by a positive HIV-1 RNA test: at approximately one week (median: 4 days), one month (median: 32 days) and six months (median: 170 days); this diagnosis occurred a median of four days after the last negative test [8]. Approximately ten HIV-1 genomes were derived via endpoint-dilution from plasma samples for each participant at each time point, yielding about thirty NFL genomes per participant (total = 761 NFL genomes, 379 5' half genomes, 416 3' half genomes and 81 additional env sequences).'

L237: The word "skyline" here is extraneous and a little confusing.
We agree and have removed it.

L309-310: Could you calculate overlapping coefficients for the dates estimated using different founder populations from the same individual?
Thanks for the suggestion. We updated Figure 7 to show the posterior distributions for each founder population and added the overlap coefficient to the accompanying table for those participants where we could analyze two subpopulations. We also added the following to the text:

'Within a single participant, estimates tended to be disparate, and were more than 30 days apart in five out of nine cases where two subpopulations were analyzed, with very little overlap between the posterior distributions (Figure 7). However, two participants, 10220 and 30812, had concordant estimates between subpopulations (overlap coefficient > 0.7), which fell only a few days prior to diagnosis.'

Whilst re-doing this figure, we also found an error in the number of polymorphic sites printed for some founder populations; this has now been fixed.

L370-371: I think this is not clearly worded enough, perhaps "the common ancestor of the sampled sequences existed a considerable time after the point of infection"? This issue is also not discussed in the context of multiple founder strains, where it is also an explanation for disagreement in estimates.
We thank the reviewer for the suggested phrasing and have changed this sentence accordingly. We have also edited the sentence referring to this issue in the discussion about multiple founders to make the connection with the earlier discussion more explicit. It now reads:
'For four participants infected with multiple founders, at least one subpopulation estimate gave dates after diagnosis, suggesting that the common ancestor of those sequences occurred much more recently than the founding infection; given the small sample size (as discussed above), it is likely that

this set of sequences represented a subpopulation that diverged recently (from a large viral population corresponding to a chronic infection).'

L393-394: What evidence of recombination was found, and how? Also, since recombination could make phylogenetic inference unreliable, simply adding more sequences will not solve the problem. It would need to be checked for explicitly. Could recombination potentially explain the too-distant TMRCA estimates for some founder strains in 10203, 20337, 30124 and 40123? If single-gene analyses for the same samples did not behave this way I would strongly suspect it. Or are those long branches themselves the evidence?
Thank you for highlighting this omission - we used both RDPv4.83 and RAPR to look for signals of recombination, and we have now cited these software in the text. We agree that recombination could potentially explain the more distant results for the participants the reviewer highlighted, as smaller effects were seen for the *env* results in Rolland et al (2020, *PLoS Path*). The effect of recombination on estimates likely depends on how deep in the tree were the ancestral branches on which the recombination events occurred, how heterogeneous the parents lineages were, and where in the genome it happened. We have updated the discussion, adding that additional sequences would help obtain a sufficiently large dataset after putative recombinants have been removed:

'Besides the small number of sequences per participant, recombination across sequences is also likely to negatively impact estimates. The combination of peak viral load and depletion of target CD4 cells that happens in acute infection means that recombination is likely frequent. While it was not an issue for the homogeneous infections with single founders, we found evidence of recombination across the different populations in infections with multiple founders [27,28]. Recombination can distort branch lengths and cause false signals of exponential growth [29], which could explain the discrepancies between subpopulations (due to the small number of sequences we did not remove recombinants when defining populations, since sufficiently distant recombinant lineages might be expected to cluster into their own subpopulation). Fitting a relaxed clock model may help control these, effectively assuming that a recombination event on a branch is equivalent to that branch having a relatively high mutation rate relative to branches without recombination events. To reduce the possible effects of recombination, we recommend increasing the sequencing depth at each time point, to obtain a sufficiently large dataset to capture and analyze each founder subpopulation separately after having removed all putative recombinants.'

L427-431: I don't think this is worded strongly enough. If the authors agree that the use of the birth-death model is inadvisable, I recommend removing it from the manuscript and adjusting the results accordingly.
We consider that it is worthwhile noting that the birth-death model could offer a useful framework for understanding within-host dynamics, and it performed remarkably well despite our choice to use non-informative priors, thus we decided to retain these results in the manuscript. We added the following to the Discussion and also added a sentence to the methods as requested by Reviewer 3.

'The birth-death model is a forwards-in-time model designed for the speciation and extinction of lineages at the population level, and has traditionally been applied to between-host epidemiological models of virus dynamics, rather than within-host. While the birth-death model could potentially offer a useful framework for the transition from a localized infection to a systemic one, we did not evaluate this here. Still, it performed surprisingly well, being selected in the best-fitting model combination for the majority of participants infected with a single founder for *gag* (62%) and typically giving a date of infection within one week prior to diagnosis (median: 5.2; range: 0.16-9.5 days prior to diagnosis). It

was selected less frequently for *env* and *pol*, but with similar overall accuracy. This suggests it can be adapted to the within-host setting, but caution is recommended, especially in the selection of priors.'

Figure 4: This looks worse for the authors than I think it needs to. Perhaps add another shaded area representing plausible eclipse periods?
We thank the reviewer for this suggestion and added a shaded box to highlight the period between 7 and 14 days prior to diagnosis.

Reviewer #3:

The authors have sufficiently addressed the comments and have made significant adjustments to the manuscript to reflect the answers.

With some very minor remaining comments I think that the manuscript is in a good shape for publication.

Minor comments:
Line 109: From what I understand now there are 30 NFL genomes per 39 participants, which should come to 1,170 sequences in total. Where do the other 110 sequences come from? Or is it that there are not exactly 10 NFL genomes per time point per patient but sometimes randomly a few more?
We revised the text as this discrepancy was also pointed by Reviewer 1, see the response to the first comment from Reviewer 1 above.

Lines 231-233: This sentence is slightly different from the version in author answers and that small change makes it very confusing. Suggested change:
"All participants had at least one model combination including a skyline in the top three ranked models, with 14 out of 25 participants having the skyline population model for the top three models selected."
to
"All participants had at least one model combination including a skyline among the top three ranked models, while 14 out of 25 participants had the skyline population model for all the top three models selected."
We thank the reviewer for catching this discrepancy. We have changed the text as suggested.

Line 275: What do "better results" mean in in this context? More precise, more accurate or more realistic maybe? Using a different comparator rather that "better" would make this title clearer.
We agree, this could have been phrased more clearly. We used the adjective 'realistic' and changed the title of this section to 'Splitting multiple founders into founding subpopulations gave more <u>*realistic*</u>, but not robust, results'. We also edited a sentence in the second paragraph of this section that also used 'better', it now reads: 'The estimates for *gag* and *pol* were <u>*closer to diagnosis*</u>, but still implausible given our knowledge on the recency of the infections…'

Line 283: Could there be a typo in "17,127 days"? Or did the authors actually get point estimates for infection at 46.5 years prior to diagnosis?
This is not a typo. The best fitting model for participant 30812 was the strict clock with skyline population model, which converged with a very low estimated evolutionary rate (5.86×10^{-7}

subs/site/year). This effectively pushed the root of the tree deep back in time, giving an estimated TMCRA of 17,127 days prior to diagnosis (95% HPD 865-301,327). The estimates from the other model combinations were less extreme, but still implausible – e.g. the UCLD relaxed clock with constant population model was ranked second and gave an estimate of 158 days prior to diagnosis (95% HPD: 28-407).

Line 523: Here I would greatly appreciate the adding of a similar comment as was given to my question on priors. Leaving priors on default values is a very dangerous approach which should be discouraged, as mostly the priors make no sense and are only there such that the software would not crash. The authors selected such priors on purpose and I think that stating that extremely clearly will minimise the risk of someone copying these analyses without thinking this over. While this is mentioned in the discussion now, I think that reiterating the reasoning here is very important.
We have added the following to the methods:
'The main concern with this dataset was that we were at the lower limit in terms of information content for these datasets given that a majority of sequences were sampled in the first month after diagnosis, especially when considering the short genes in the HIV-1 genome. Therefore, we generally opted for non-informative priors with the rationale that sufficient data will overwhelm a non-ideal choice of prior and still produce reasonable posterior distributions. In this way, we could determine instances when our sequence data were not informative for parameter estimation.'
